# Exploratory Profiling of Extracellular MicroRNAs in Cerebrospinal Fluid Comparing Leptomeningeal Metastasis with Other Central Nervous System Tumor Statuses

**DOI:** 10.3390/jcm10214860

**Published:** 2021-10-22

**Authors:** Ji Hye Im, Tae Hoon Kim, Kyue-Yim Lee, Ho-Shin Gwak, Weiwei Lin, Jong Bae Park, Jong Heon Kim, Byong Chul Yoo, Seong-Min Park, Ji-Woong Kwon, Sang Hoon Shin, Heon Yoo

**Affiliations:** 1 Department of Cancer Control, Graduate School of Cancer Science and Policy, National Cancer Center, Goyang 10408, Korea; 75262@ncc.re.kr (J.H.I.); 70564@ncc.re.kr (K.-Y.L.); 2 Department of Cancer Biomedical Science, Graduate School of Cancer Science and Policy, National Cancer Center, Goyang 10408, Korea; 71917@ncc.re.kr (T.H.K.); 97055@ncc.re.kr (W.L.); jbp@ncc.re.kr (J.B.P.); jhkim@ncc.re.kr (J.H.K.); yoo_akh@ncc.re.kr (B.C.Y.); lastmhc@ncc.re.kr (S.-M.P.); heonyoo@ncc.re.kr (H.Y.); 3 Neuro-Oncology Clinic, National Cancer Center, Goyang 10408, Korea; jwkwon@ncc.re.kr (J.-W.K.); nsshin@ncc.re.kr (S.H.S.); 4 Cancer Molecular Biology Branch, Division of Cancer Biology, Research Institute, National Cancer Center, Goyang 10408, Korea; 5 Division of Translational Science, Research Institute, National Cancer Center, Goyang 10408, Korea

**Keywords:** leptomeningeal metastasis, cerebrospinal fluid, microRNA, microarray, biomarker

## Abstract

The diagnosis of leptomeningeal metastasis (LM) is often difficult due to the paucity of cancer cells in cerebrospinal fluid (CSF) and nonspecific findings on neuroimaging. Investigations of extracellular microRNAs (miRNAs) in CSF could be used for both the diagnosis and study of LM pathogenesis because they reflect the activity of disseminating cancer cells. We isolated CSF extracellular miRNAs from patients (*n* = 65) of different central nervous system tumor statuses, including cancer control, healthy control, LM, brain metastasis (BM), and primary brain tumor (BT) groups, and performed miRNA microarrays. In unsupervised clustering analyses, all LM and two BM samples showed unique profiles. Among 30 miRNAs identified for LM-specific biomarkers via a Prediction Analysis of Microarrays, miR-335-5p and miR-34b-3p were confirmed in both the discovery and validation samples (*n* = 23). Next, we performed a significance analysis of the microarray (SAM) to extract discriminative miRNA profiles of two selected CSF groups, with LM samples revealing a greater number of discriminative miRNAs than BM and BT samples compared to controls. Using SAM comparisons between LM and BM samples, we identified 30 upregulated and 6 downregulated LM miRNAs. To reduce bias from different primary cancers, we performed a subset analysis with primary non-small cell lung cancer, and 12 of 13 upregulated miRNAs in LM vs. BM belonged to the upregulated miRNAs in LM. We identified possible target genes and their biological processes that could be affected by LM discriminative miRNAs in NSCLC using the gene ontology database. In conclusion, we identified a unique extracellular miRNA profile in LM CSF that was different from BM, suggesting the use of miRNAs as LM biomarkers in studies of LM pathogenesis.

## 1. Introduction

Cerebrospinal fluid (CSF) circulates throughout the entire neuroaxis and bathes the central nervous system (CNS). Various cells residing in the CNS secrete biological constituents and bioactive substances including proteins, metabolites, and RNAs, and some micro-molecules from the systemic circulation penetrate into the CSF [1,2]. Utilizing the biomolecules in CSF as biomarkers of CNS diseases could be useful because it is difficult to diagnose and monitor these diseases directly based on tissue biopsies [3,4,5]. Leptomeningeal metastasis (LM) involves terminal-stage cancer progression, characterized by cancer cells spreading through the CSF space; however, without predictable markers and effective treatments, the overall survival for patients is only approximately 6–8 weeks [6]. The pathogenesis of LM is less studied but likely differs from that of other CNS tumors or brain metastases [7]. LM cancer cells must adapt to an under-nutritious environment with low levels of nutrients and growth factors in the CSF, when compared to the parent cancer cells, which utilize serum as their resources [8,9,10,11,12,13]. The pathogenesis of LM remains unknown, and the therapeutic options for LM patients are also limited. Thus, characterizing LM pathology is important in the development of effective treatments for this disorder.

MicroRNAs (miRNAs), which are non-coding RNAs with approximately 22 nucleotides in length, can regulate mRNA expression post-transcriptionally in numerous biological processes, including as development and differentiation [14]. Recent studies show that miRNAs can reflect the behavior of cells in cancer progression, such as during malignant transformation and cancer metastasis. However, it is difficult to obtain miRNA profiles of various CNS tumors from CSF samples because of their very low concentrations in CSF compared to serum. Normal CSF rarely contains floating cells unless the patient has a CNS disease. Even in cases of LM, at which cells in CSF were enough to be precipitated for cytology examination, the cell population is composed of cancer cells and immune cells in approximately half-and-half [15]. No study has succeeded in separating cancer cells from immune cell populations in CSF at a sufficient quantity to perform genomic analyses, such as single-cell sequencing or microarray, from individual CSF samples of 2‒10 mL.

Extracellular miRNAs are believed to act as messengers between cells and the microenvironment, and miRNAs in biofluids could be useful as biomarkers obtained from a liquid biopsy to predict tumor burden, as well as the patient’s prognosis, in the clinic [16,17,18]. Previous studies show that relatively low concentrations and small numbers of detectable extracellular miRNAs could be isolated from the CSF [19,20,21]. Other studies reported that CSF extracellular miRNAs are differently expressed in CNS diseases [22,23,24]. However, it is hard to confirm certain extracellular miRNAs in CSF as biomarkers for LM, and to correlate miRNA profiles with supportive multi-omics data such as CSF proteomics. Furthermore, because the source of extracellular miRNAs is variable, especially in LM, including cancer cells, immune cells, and brain cells, we assumed that a comparison between the two appropriately selected groups would reflect the profiles representing certain cells or the environment (i.e., discriminative miRNAs of systemic cancer CSF compared with healthy controls reflecting a diffusion of miRNAs from cancer cells in systemic circulation into CSF).

Here, we extracted extracellular miRNAs from 2 mL (clinically accessible) CSF samples of individual patients for miRNA microarray analysis. Considering that miRNAs come from various sources of CNS constituents and are affected by many variables, we hypothesized that the direct profiling of LM samples compared to control samples might fail to reflect LM characteristics but could represent other elements, such as the systemic cancer status or the accompanied CNS parenchymal tumor. Thus, by comparing miRNA profiles between different CNS tumor statuses of the non-CNS cancer control (CC), healthy control (HC), LM, brain metastasis (BM), and primary brain tumor (BT), we could extract miRNA profiles representing characteristics to be used for comparative analyses between appropriately selected groups. For example, discriminative of miRNA profiles of BM compared with BT could reflect the process of metastasis as BM and BT have the same condition of a parenchyma space occupying a lesion of the brain. In addition, the discriminative miRNA profile of LM compared with BM could represent the uniqueness of floating cancer cells as LM and BM have the same characteristics of a metastatic lesion to CNS.

We therefore characterized the differences of miRNA profiles between two selected groups using a significance analysis of the microarray (SAM) and adopted prediction analyses for microarrays (PAM) to define representative miRNAs of LM samples. Selected discriminative miRNAs were examined by the digital droplet polymerase chain reaction (ddPCR) using external validation samples independent from the microarray data. In addition, we analyze possible target genes of LM discriminative miRNAs using gene ontology (GO) pathway analysis.

## 2. Materials and Methods

**Figure 1:**
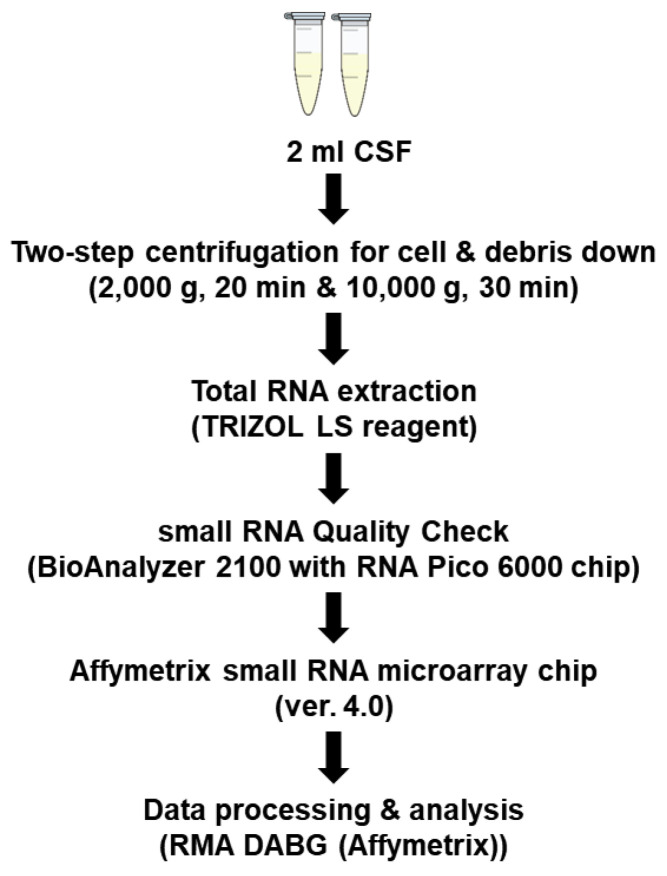
The workflow of cerebrospinal fluid microRNA microarray experiments.

The extracellular miRNA extraction from individual samples, quality control, and the microarray profiling workflow are summarized in Figure 1.

### 2.1. CSF Preparation and Archives

The CSF samples of patients who previously submitted informed consent were obtained for this study after approval by the Institutional Review Board (Identifier: 2014-0135).

The CSF sampling site was varied among individual patients and groups according to the specific experimental purpose. For CC patients, CSF samples were obtained via a lumbar puncture during spinal anesthesia, and for HC subjects, CSF was obtained from the cisternal/subarachnoid space during brain retraction on a craniotomy for unruptured aneurysms or diagnostic lumbar puncture for hydrocephalus. In patients with CNS tumors (LM, BM, and BT), CSF was obtained during a CSF cytology examination via lumbar puncture, from the ventricle after an Ommaya reservoir placement, the cisternal/subarachnoid space during craniotomy for tumor removal, or therapeutic drainage for controlling either increased intracranial pressure (ventricular) or CSF leakage (lumbar) (Table 1). The CSF sample was centrifuged (2000× *g* for 20 min) within 1 h of collection to pellet the cells, and the supernatant was aliquoted and stored overnight at −20 °C or immediately centrifuged at 10,000× *g* for 30 min to remove cell debris and kept frozen at −80 °C until being used for RNA extraction.

### 2.2. Definition of Patient Groups

To define CC patients, recent computed tomography or positron-emission tomography (PET) images (≤3 months) or recent bone marrow reports were evaluated for existing cancer lesions at the time of CSF sampling. All LM patients had both a cytological diagnosis of LM and positive neuroimaging study (gadolinium-enhanced brain magnetic resonance imaging (MRI)/whole-spine MRI) results [25]. All BM and BT patients were pathologically confirmed at or after CSF sampling using surgical specimens, and the presence of brain tumors at the time of sampling was also verified by MRI. To avoid bias from concurrent BM in patients with LM, we carefully selected LM samples without parenchymal BM to be included in this study. Because the CC, BM, and BT groups were at a high risk for developing LM, all CSF samples were examined negative of LM by CSF cytology, and patients with less than a 3-month follow-up after CSF sampling were excluded from the study.

### 2.3. Nucleic Acid Extraction and Quantification

Extracellular RNA was extracted from 2 mL of the above pre-centrifuged CSF using TRIzol LS reagent according to the manufacturer’s instructions (Thermo Fisher Scientific, Waltham, MA, USA). Briefly, we mixed 1 mL TRIzol LS Reagent with 500 μL CSF and added 270 µL chloroform; the homogenate would be separated into 3 layers. For RNA precipitation, we used 670 µL 100% isopropanol to the upper clear aqueous layer, which contained RNA, followed by incubation at room temperature for 10 min. The samples were then centrifuged with 12,000 × *g* at 4 °C for 10 min. For the washing step, 1 mL 75% ethanol was used, and the mixture was centrifuged for 5 min at 7500× *g* at 4 °C. After discarding the supernatant and air drying the RNA pellet for 5–10 min, the pellet was resuspended with 20–50 µL of RNase-free water and incubated in a heat block at 55 °C for 10–15 min. The dissolved RNA samples were measured for purity (A_230_/A_280_ ratio) and the concentration using Nanodrop spectrophotometers (ThermoFisher Scientific, Waltham, MA, USA).

### 2.4. miRNA Microarray

The quantity and quality of extracted miRNAs were determined using the Agilent BioAnalyzer 2100 with a smallRNA chip (Agilent Technologies, Columbia, MD, USA). The miRNA yields were normalized relative to a nanogram of RNA per milliliter of CSF. RNA was labeled with an Affymetrix FlashTag Biotin HSR RNA Labeling Kit (Affymetrix, Santa Clara, CA, USA) according to standard procedures. Labeled RNA was hybridized to Affymetrix GeneChip miRNA 4.0 arrays, and then analyzed using the Fluidics Station 450 protocol (FS450_002, Affymetrix). The array was designed to study a total of 6599 small non-coding RNAs (2578 mature miRNAs, 2025 pre-miRNAs, and 1996 small nucleolar RNAs). Affymetrix CEL files were processed in the Affymetrix Expression Console using the robust Multi-chip Analysis (RMA) method. Raw data were deposited into the NCBI’s Gene Expression Omnibus (https://www.ncbi.nlm.nih.gov/geo, accessed on 29 September 2020) according to GEO guidelines (GSE number 138092).

### 2.5. Synthesis of miRNA cDNA and ddPCR

Approximately 2 ng of purified total RNA was reverse transcribed to generate cDNA using a TaqMan Advanced miRNA cDNA Synthesis Kit (A28007, Applied Biosystems, Foster City, CA, USA) according to the manufacturer’s instructions. Four microliters of cDNA were mixed with the ddPCR Supermix for Probes (no dUTP, Bio-Rad Laboratories, Hercules, CA, USA) and TaqMan Advanced miRNA Assay probes (Applied Biosystems; hsa-miR-466, hsa-miR-34b-3p, hsa-miR-335-5p). Four microliters of cDNA were mixed with the QX200 ddPCR EvaGreen Supermix (Bio-Rad Laboratories, Hercules, CA, USA). Each 20 µL reaction mixture was mixed with a 70 µL droplet generation oil and partitioned in up to 20,000 nanoliter-sized droplets using the QX299 droplet generator (Bio-Rad Laboratories, Hercules, CA, USA). Finally, 40 µL of the droplet mixture was used for PCR with the following cycling protocol: 95 °C for 5 min (DNA polymerase activation), followed by 40 cycles of 95 °C for 30 s (denaturation) and 55 °C for 1 min (annealing) followed by post-cycling steps of 98 °C for 10 min (enzyme inactivation) and an infinite 4 °C hold using the 7900HT Sequence Detection System (Applied Biosystems). Cycling between the temperatures was set to a ramp rate of 2.5 °C/s. The amplified PCR products of the nucleic acid targets in the droplets were quantified in the FAM channels using a QC200 Droplet Reader (Bio-Rad Laboratories) and analyzed using QuantaSoft version 1.7.4.0917 software (Bio-Rad Laboratories, Hercules, CA, USA). At least 15,000 drops per well were analyzed, and samples with fewer than 15,000 drops per well were repeated via ddPCR. The concentration (miRNA copies/μL) values generated by QuantaSoft were converted to miRNA copies/nL of CSF.

### 2.6. miRNA Profiling and Functional Enrichment Analysis

Differentially expressed miRNAs were identified according to patient groups using SAM or PAM [26]. Using SAM passive diffusion of miRNAs from blood vessels into CSF was analyzed by comparing the miRNA expression levels between control samples (CC and HC). Next, we investigated differentially expressed miRNAs in patients with CNS tumor conditions (LM, BM, and BT) when compared with controls (CC and HC). Finally, we compared miRNA expression profiles in CSF samples from patients with LM and BM. The process was initialized with 200 permutations, the K-Nearest Neighbor imputation algorithm, and S0 parameters selected by Tusher et al.’s method using the samr package [27]. Predicted target genes of miRNA were searched from miRTargetLink Human [28], and a GO enrichment analysis of target genes was performed from the Database for Annotation, Visualization, and Integrated Discovery (DAVID) [29,30].

### 2.7. Statistical Analysis

Categorical data were analyzed using the Chi-square, Fisher’s exact, or Mann–Whitney *U* tests as appropriate. Quantitative data were analyzed using a one-way analysis of variance with post-hoc comparisons (Scheffe’s test). Statistical significance was determined using Student *t*-tests, and a *p*-value < 0.05 was considered to indicate a statistically significant result. The statistical analysis and data visualization were performed using the ROCR, stats, and ggplot2 package of studio R (ver. 3.6.0), and GraphPad Prism 3.0 (San Diego, CA 92108, USA).

## 3. Results

### 3.1. Clinical Characteristics of Patients and CSF

The demographic characteristics of individual patients are presented in Table 1. We enrolled 65 patients for the discovery set, 23 patients for the external validation set, and CSF samples for grouping by different CNS tumor statuses. In the discovery set, the median age was 56 years (range, 2.3–78 years). The CC group patients (*n* = 11), including four with leukemia, had systemic cancer at the time of CSF sampling. The HC group (*n* = 12) was mostly patients with unruptured cerebral aneurysms diagnosed at medical examinations, whose CSF was sampled at the time of the craniotomy for aneurysm clipping. The most frequent primary cancer of the LM group was lung cancer (*n* = 10), followed by breast cancer (*n* = 5) and ovarian cancer (*n* = 2). The BM group (*n* = 11) included patients with primary lung cancers, melanomas, breast cancers, and hepatocellular carcinoma. The BT group (*n* = 9) included patients with gliomas, schwannomas, and other brain tumors.

### 3.2. Yield and Quality of CSF Extracellular RNA

The mean RNA yield of all patients was 7.31 μg (range 2.80–26.5 μg), determined using the Nanodrop 1000 spectrophotometer (Supplementary Table S1 and Supplementary Figure S1A). The mean RNA yield of LM samples was significantly higher than that of other groups (9.28 vs. 6.30 μg, *p* < 0.01). The percentage of the small RNA fraction (<150 nucleotides) was 35% (range: 1–69%) for all patients, calculated using the Agilent BioAnalyzer 2100 (Agilent Technologies). The non-CNS tumor groups (CC and HC together) had a relatively higher percentage of small RNA than other groups (53% vs. 25%, *p* < 0.001; Supplementary Figure S1B). The RNA yield of several samples having a reverse was confirmed using the RiboGreen RNA assay (Thermo Fisher Scientific), which provided quantitation based on fluorescence specific for single-stranded RNA. The concentration of RNA measured by the RiboGreen RNA assay was a mean of 56.7 ng/μL (range; 5–245), and it was 0.14-fold of that from the NanoDrop spectrophotometer (mean 3313.5 ng/μL, range; 36–54891) when measuring the same samples (Supplementary Figure S1C; *n* = 8). Therefore, we could estimate that there was contamination in our RNA extracts (probably degraded DNA), as real amounts of small RNA were calculated from this fold difference to be 1.02 ng/μL (range; 0.39–3.71).

### 3.3. Hierarchical Clustering of Mature miRNAs Can Differentiate LM from other CNS Tumor Groups

**Figure 2:**
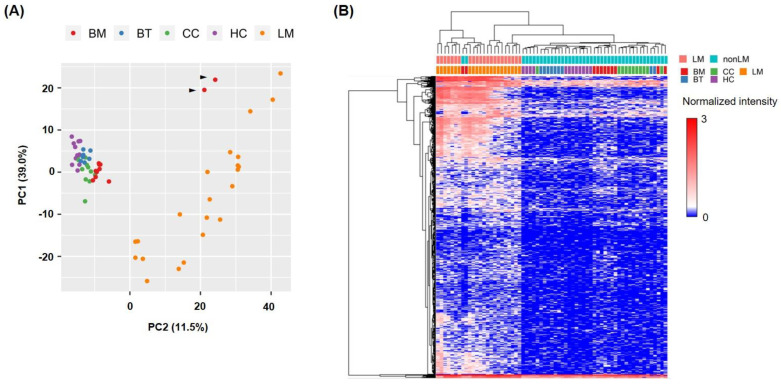
CSF miRNA profiling can distinguish the leptomeningeal metastasis group from the other CNS tumor groups. (**A**) Principal component analysis (PCA) of the microarray data. Black arrows indicate two BM samples. (**B**) Unsupervised hierarchical clustering analysis. A total of 998 mature miRNAs (group FDR F-test, *p* < 0.05) were selected, and k-means clustering method was used to generate dendrograms. Abbreviations: BM, brain metastasis; BT, brain tumor, CC, cancer control; HC, healthy control; LM, leptomeningeal metastasis.

We identified the extracellular miRNAs differentially expressed in the CSF of patients. The raw data files are available in Gene Expression Omnibus (GSE138092). To summarize the microarray data, we performed a principal component analysis (PCA) and unsupervised hierarchical clustering analysis (HCA). In the PCA plot, a heterogeneous profile of the LM group was identified, while other groups had similar profiles except two BM samples (Figure 2A). In the HCA, two major clusters of differentially expressed miRNAs separated the LM samples from the other samples, except the same two BM samples (Figure 2A,B). One patient of the two BM samples failed to meet the diagnostic criteria of LM at the time of CSF sampling with negative of the CSF cytology but suspicious sulci involvement on MRI. However, in a follow-up, MRI revealed ventriculomegaly, which implied a suggestive LM finding, but the patient was lost to a further follow-up. Another patient had cerebellar hemorrhagic metastasis, which had a high risk of LM development, but the follow-up MRI was not performed. Together, these results suggested that the miRNA profiling may differentiate true LM, as well as a high risk of LM from non-CNS tumors or other CNS tumors.

### 3.4. PAM Differentiated LM Patients LM from other Groups

**Figure 3:**
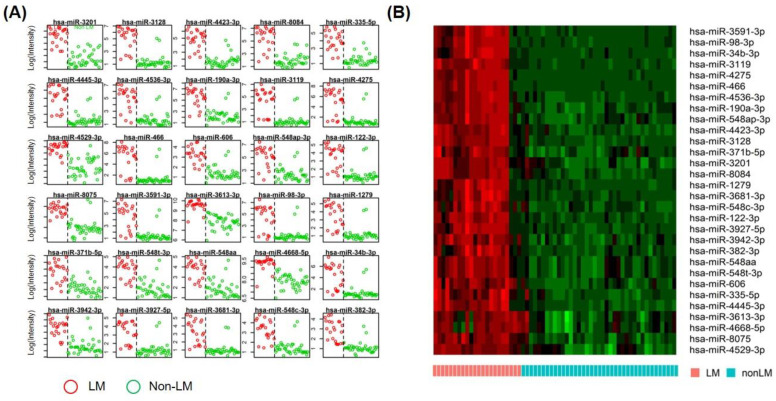
A prediction analysis of microarray (PAM) revealed 30 discriminative extracellular microRNAs of LM CSF compared to other patient groups (red dots are from LM and green dots from other groups). (**A**) Gene expression plot of 30 miRNAs represented LM and other groups’ miRNA expression. (**B**) The heatmap of 30 miRNAs represented miRNAs’ expression, and the tile plot showed LM classification of PAM analysis.

Based on the PCA and HCA results, we hypothesized that the miRNA profile of LM was significantly different from that of other CNS tumor groups. To identify miRNAs characteristic of LM, we performed PAM, an algorithm that generates a minimal gene set to characterize groups based on the nearest shrunken centroids of gene expression [26,27]. Because four samples (two of BM and two of BT) failed to fulfill the spike-in control threshold, we used 61 samples to conduct the PAM algorithm. After PAM analysis, we selected a list of 30 miRNAs with a minimum classification error (6/61, 9.8%). These miRNAs were used to predict LM samples (Figure 3A,B). These miRNAs included miR-3201, miR-3128, miR-4423-3p, miR-8084, miR-335-5p, miR-4445-3p, miR-4536-3p, miR-3119, miR-4275, miR-466.

### 3.5. SAM and Annotation of Discriminatively Expressed miRNAs between Different Patient Groups

To address individual miRNA differences between groups, we performed SAM with a false discovery rate (FDR) < 0.10 [27].

**Figure 4:**
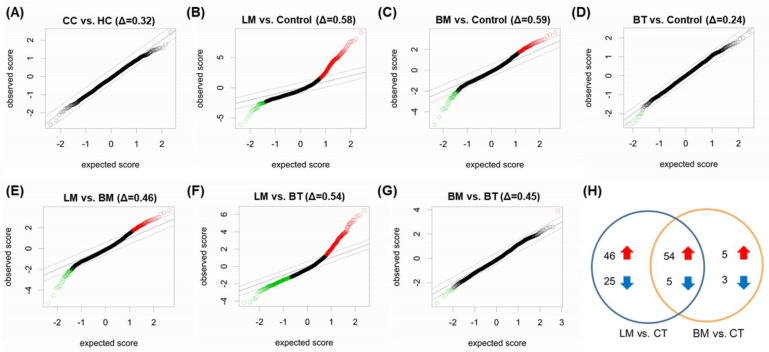
Significance analysis of microarrays shows different expression levels of miRNAs between patient groups. (**A**) HC and CC, (**B**) LM and Controls (HC + CC), (**C**) BM and Controls, (**D**) BT and Controls, (**E**) LM and BM, (**F**) LM and BT, and (**G**) BM and BT. (**H**) The Venn diagram showed numbers of miRNAs that differentially expressed in LM and BM samples. The intersection represents common miRNAs. The red arrow indicates upregulated miRNAs and the blue arrow indicates downregulated miRNAs. Delta values at a 10% false discovery rate are presented at the top of each graph. Abbreviations: BM, brain metastasis; BT, brain tumor, CC, cancer control; HC, healthy control; LM, leptomeningeal metastasis.

First, because it is well-known that biofluids of cancer patients contain various miRNAs from cancer cells [8], we compared HC samples with CC samples to verify whether miRNAs from systemic circulation could affect CSF extracellular miRNA profiles. SAM between the HC and CC samples showed that there were no differentially expressed miRNAs with >2-fold change (FC) in expression (Figure 4A), but the expressions of six miRNAs were lower in the CC samples than in the HC samples with a FC ranging from 0.62 to 0.79 (Supplementary Table S2). Based on this result, we combined the HC and CC samples with the “control (CT)” samples for further analysis.

Second, we determined whether the CNS tumor groups had different CSF miRNA profiles compared to control samples (Supplementary Table S2). CSF samples from LM patients contained 108 differentially expressed miRNAs (>2 FC), with 100 miRNAs being upregulated, and 8 miRNAs being downregulated in LM patients compared with CT subjects (Figure 4B). The top 10 upregulated and downregulated miRNAs in LM samples are listed in Table 2 (left column). The representative miRNAs upregulated in LM patients with a FC ranging from 6.81 to 15.4 included miR-466, miR-98-3p, miR-34b-3p. Downregulated miRNAs in LM patients had FCs ranging from 0.29 to 0.49 and included miR-455-3p, miR-3921, miR-1298-3p. SAM between BM and CT identified 59 upregulated miRNAs and 30 downregulated miRNAs in BM samples (Figure 4C). The top 10 upregulated miRNAs in BM samples with FCs ranging from 4.40 to 12.3 included also miR-466, miR-98-3p, miR-34b-3p (Table 2, middle column). The top 10 downregulated miRNAs in BM samples with FCs ranging from 0.18 to 0.38 included miR-1298-3p, miR-628-5p, miR-877-5p. In addition, the algorithm between BT and CT samples showed no highly expressed miRNAs but only two suppressed miRNAs (Figure 4D, Table 2, right column). Together, these results suggest that primary BT minimally affected the extracellular miRNA profile of CSF, whereas LM patients, in whom tumor cells were actively proliferating in CSF, had the highest differential expressions of extracellular miRNAs.

We then analyzed the differentially expressed miRNAs between CNS tumor groups (Supplementary Table S2). SAM revealed 30 upregulated and 6 downregulated miRNAs in LM samples compared with BM samples (Figure 4E). When we performed SAM between LM and BT samples, 101 miRNAs were upregulated, and 11 miRNAs were downregulated in LM samples (Figure 4F). SAM between BM and BT samples identified one upregulated miRNA, whereas 29 miRNAs were downregulated in BM samples (Figure 4G).

We then estimated the similarities of differentially expressed miRNAs in LM (compared to CT samples) with those of BM and BT, respectively. As the BT group showed no upregulated but only two downregulated miRNAs compared to CT, we saw no common differentially expressed miRNA between LM and BT samples, except one downregulated miRNA, miR-4708-3p. However, most of the differentially expressed miRNAs in BM samples were up- or downregulated in LM samples (Figure 4H). Fifty-four of 59 (92%) upregulated and five of eight (63%) downregulated miRNAs in BM samples were also differentially expressed in LM samples. Thus, we suggest that LM samples shared most of the characteristic miRNAs of BM samples, which could possibly result from the interaction between metastatic tumor cells and the host brain, and differentially expressed miRNAs of LM compared with BM samples could possibly be acquired from the activity of floating cancer cells.

### 3.6. Identification of Highly Expressed miRNAs in LM Samples Compared with BM Samples in Primary Non-Small Cell Lung Cancer (NSCLC)

We assumed that the comparison between LM and BM samples could identify miRNA profiles reflecting cancer cell adaptation from the brain parenchyma to aqueous CSF environments. However, the limitation of our analysis was that we could not adjust for primary cancers, which are known to affect miRNA profiles. To minimize the bias of miRNA profiling according to different primary cancers, we performed a subset analysis of primary NSCLC (16 from LM and 5 from BM patients), and compared those miRNAs to miRNAs which were significantly differentially expressed in SAM between all LM and BM samples (Table 3). In this comparison, 12 out of 13 (92%) upregulated (FC > 2) miRNAs in NSCLC LM were noted in the list of upregulated miRNAs in all LM compared with all BM.

### 3.7. Confirmation of Discriminative miRNA Expression in LM Samples by ddPCR

**Figure 5:**
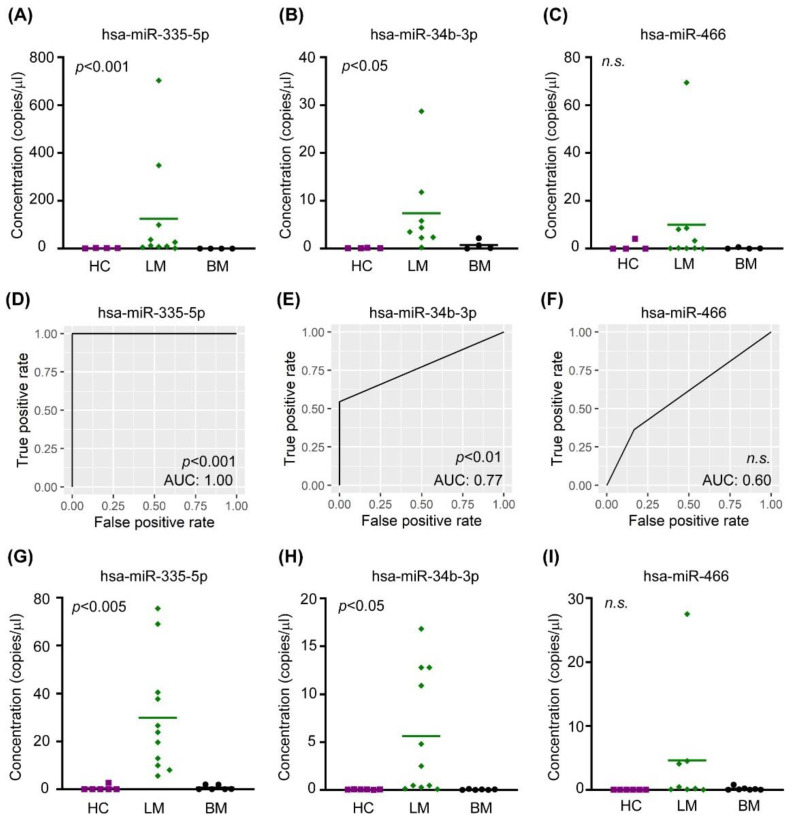
Internal and external validation of the representative discriminative miRNAs by digital droplet PCR (ddPCR) with corresponding analysis of Area Under the Receiver Operating Characteristics (ROC) for LM. (**A**–**C**) Expressions of indicated miRNAs were determined through ddPCR in the discovery sample set (HC *n* = 4, BM *n* = 4, LM *n* = 8–10). Two sample *t*-test *p*-values between LM and non-LM were calculated in Prism software. (**D**–**F**) The area under the ROC of indicated miRNA was calculated using R software (version 3.6.0). (**G**–**I**) Expressions of indicated miRNAs were determined through ddPCR in the validation sample set (HC *n* = 6, BM *n* = 6, LM *n* = 11). Bar represents mean value. Abbreviations: HC, healthy control; LM, leptomeningeal metastasis; BM, brain metastasis.

To validate microarray data, we selected three discriminative miRNAs of LM patients based on both PAM and SAM analyses, and the expression levels of theses miRNAs were confirmed by ddPCR. In the discovery set, the miR-335-5p, miR-34b-3p, and miR-466 expression levels were compared among 8 LM samples, 4 BM samples, and 4 HC samples (Figure 5A–C). The mean concentrations of three miRNAs were higher in LM samples, when compared with non-LM samples, and statistical significances were found for miR-335-5p and miR-34b-3p (*p* <0.001 and <0.05, respectively, two-sample *t*-test). Moreover, we calculated the threshold of the ddPCR concentration in which the classification scores were obtained (Supplementary Figure S2A–C).

Additionally, we enrolled 23 CSF samples (6 of HC, 6 of BM, and 12 of LM), which were not used in the microarray analysis, as a validation set and performed ddPCR (Figure 5G–I). The mean concentrations of three miRNAs were highest in the LM samples, and statistical significance was found for miR-335-5p and miR-34b-3p (*p* < 0.005 and *p* < 0.05, respectively, two-sample *t*-test). At the ddPCR thresholds calculated, miRNAs predicted LM samples with better classification scores than the discovery set. We confirmed the diagnostic performance of miRNAs using a receiver operating characteristic (ROC) curve analysis. The areas under the curve of miR-335-5p, miR-34b-3p, and miR-466 were 1.00, 0.77, and 0.60, respectively (Figure 5D–F).

### 3.8. Gene Set Enrichment Analysis of Discriminatively Expressed miRNAs in LM Patients with NSCLC

**Figure 6:**
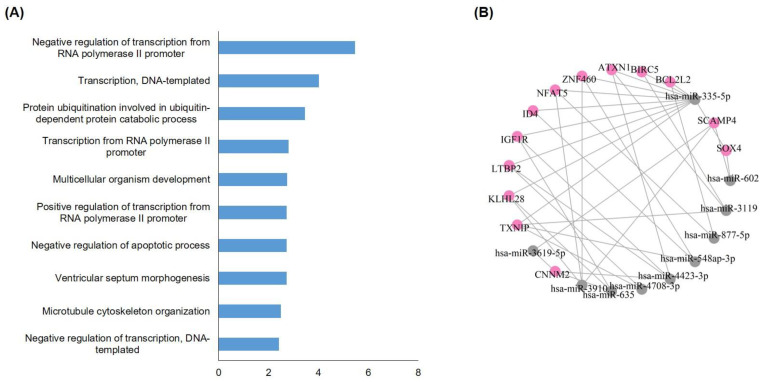
LM discriminative miRNA target gene enrichment analysis. Selected differentially expressed miRNAs between LM and BM of a primary cancer of non-small cell lung cancer predict (**A**) enriched molecular pathways by Gene Ontology, and (**B**) their target genes.

We performed GO pathway analyses to evaluate the relevance of representative miRNAs of LM samples compared with BM in the subset analysis with NSCLC samples and to verify the molecular pathways involved. First, the putative target genes of 10 upregulated and 6 downregulated miRNAs in Table 3 were searched using the miRWalk database [31] using a binding score 1.00 and a restriction of the binding site to 3′UTR. In the biological process category of DAVID GO, the most highly targeted pathway was negative-transcription from the RNA polymerase II promoter followed by a positive regulation of transcription, DNA-templated (Figure 6A). We also organized a network between 16 miRNAs and their target genes and found that some of the genes were potential targets of ≥3 miRNAs (Figure 6B). These genes were ATXN1 (Ataxin-1), CNNM2 (Cyclin M2), IGF1R (Insulin-like growth factor 1 receptor), KLHL15 (Kelch-like protein 15), LTBP2 (Latent-transforming growth factor beta-binding protein 2), NFAT5 (Nuclear factor of activated T-cells 5), SCAMP4 (Secretory Carrier Membrane Protein 4), TXNIP (thioredoxin interacting protein), ZNF460 (Zinc finger protein 460). Notably, miR-335-5p connected the network to target genes with strong evidence and a higher frequency, suggesting that the miRNA could affect many biological functions.

## 4. Discussion

This study investigated the feasibility of extracellular small RNAs microarray for profiling different CNS tumor statuses, including those with LM from a limited volume of CSF from individual patients. We successfully performed miRNA profiling for five patient groups and identified miRNA biomarkers that discriminated LM from other CNS cancers. Our results provided the basis for future studies of extracellular RNAs from individual CSF samples.

### 4.1. Extraction of Extracellular miRNA from CSF

Conventionally, the total CSF volume from an individual is approximately 150 mL so that we can obtain 5–10 mL of CSF per sampling. Monitoring CNS disease using CSF samples is challenging due to a limited sample volume and relatively invasive procedures, such as a lumbar/ventricular puncture for sample acquisition. However, an analysis of miRNAs in the CSF is expected to be better than in the serum in reflecting CNS disease activity, as CSF directly contacts with the brain. Studies reported that the CSF contains brain-specific molecules, which are not found in the serum. Yagi et al. verified differences in exosomal miRNA profiles between the CSF and serum [20]. Half of the reported brain miRNAs were found in CSF exosomal fractions, and some miRNAs specifically expressed in brain tissues were detected in CSF but not in serum. Some clinical studies with a lack of controlled comparisons with serum miRNAs reported that cancer progression was associated with levels of certain onco-miRNAs in BM and LM samples [9,23].

Because CSF is endogenously filtered through a specialized capillary called the blood-CSF barrier, amounts of its contents, including secreted miRNAs, are lower than those in the serum [32]. In addition, normally there are no floating cells in CSF, except during CNS disease. Thus, extracellular miRNA was found in CSF but at a very low concentration of 1–20 ng/mL [18]. Researchers therefore use pooled CSF samples for a microarray analysis or next generation sequencing (NGS) because more than hundreds of qualified RNA are needed to perform a valuable analysis of extracellular RNA from CSF [18,33,34]. NGS of extracellular RNA profiles in CSF was first reported by Burgo et al. in 2013 [18], who compared RNA yields using different commercially available RNA extraction kits and suggested that the resulting RNA yields of 10–30 ng per mL were too low for NGS. They then pooled all RNA extracts from 30 CSF samples of unknown disease statuses to evaluate miRNA profiles and identified 486 miRNAs in CSF. Among these, 353 (73%) miRNAs also were found in a serum control. Yagi et al. analyzed both exosomal and non-exosomal RNAs from pooled CSF samples from seven healthy volunteers [19]. Some of the top-ranked miRNAs from the study of Burgo et al. were common in the exosomal fraction (miR-204, miR-26a, miR-10a miR-10b, and miR-30a) or the non-exosomal fraction (miR-204, miR-486, miR-92, and miR-27b). Saugstad et al. tried to standardize the extraction method for extracellular RNA in CSF [33] and verified that the total RNA yield was lower in the control, Alzheimer’s disease (AD), and Parkinson’s disease (PD) compared to that in low-grade glioma (LGG) and glioblastoma (GBM). In our study, the total RNA amount was significantly higher in the LM group compared to the control samples. Because cancer cells proliferate in CSF space or the pial surface in LM, extracellular RNAs are actively secreted by LM cancer cells directly into the CSF. As a result, RNA concentrations were highest in CSF from LM patients compared with other groups. We also analyzed the miRNA fraction as a quality control, which showed that the relative percentages of miRNA were higher in the control samples than in other CNS tumor samples. This finding might suggest that CSF from the CNS tumor samples contained long RNAs, likely from disease activity in the brain parenchyma or CSF space. This relatively higher percentage of large RNA also was observed in the study of Saugstad et al. [33]. In the study by Saugstad et al., extracellular mRNA levels detected by qRT-PCR or long RNA-seq were higher in LGG and GBM than in control, AD, and PD samples, whereas the number of miRNAs did not significantly differ between the two groups. In our study, the percentages of small RNAs were lower in the CNS tumor samples compared with control samples, although we did not identify the long RNAs, which might be the result of increased mRNAs from tumor cell activity.

### 4.2. Extracellular miRNA Profiles Discriminate CNS Tumor Statuses

Studies suggest that miRNA profiles could serve as diagnostic and prognostic biomarkers for CNS diseases [35]. In neurodegenerative diseases such as Alzheimer’s dementia, amyotrophic lateral sclerosis, or PD, specific miRNAs are upregulated in both the serum and CSF [36]. The miRNA measurements are reproducible, and their profiles are significantly different, not only between normal and cancer patients but also among primary cancer types [37]. The expression of a specific miRNA (e.g., miR10b) in the serum or CSF is correlated with advanced stage or metastatic cancers [10,38]. However, the upregulation and downregulation of certain miRNAs sometimes give an opposite direction in the progression of a certain cancer types [39,40]. Teplyuk et al. performed a quantitative RT-PCR analysis of seven miRNAs including the miR-10b, miR-21, and miR-200 family, and discriminated metastatic BT from gliomas with an accuracy of >90% using machine-learning tools [41]. In the present study, we defined miRNA profiles according to different CNS tumor statuses of LM, BM, and BT patients. The unsupervised clustering of miRNAs detected by microarray revealed that LM samples had significantly more upregulated miRNAs than other groups that could be naturally clustered. LM samples also had more miRNAs that displayed a significantly different expression than other groups when using SAM analysis. In contrast, extracellular miRNAs unique to BT samples were nearly absent according to SAM, and we were unable to identify miRNA profiles in BT samples that differed from the control samples. This result may be due to the observation that approximately two-thirds of BT samples comprised benign extra-axial tumors, which typically lack an interaction with the brain or CSF, and miR expression levels in our microarray assays were at a relatively low level of amplification compared with that of the previous PCR study.

### 4.3. Extracellular miRNA Profiles Discriminate Leptomeningeal Metastasis from Parenchymal Brain Metastasis

Many studies identified candidate miRNAs that modulate cancer metastasis to the brain parenchyma according to different primary cancers, including downregulated miR-19a for breast cancer, miR-145 for lung cancers, the upregulated miR-200 family for breast/lung cancer, and miR-378 for non-small cell lung cancer [9]. These candidate miRNAs were studied in metastatic tissue rather than the extracellular fraction in biofluids. Our results showed that the supervised clustering of highly expressed extracellular miRNAs in LM was intermediate in BM compared to BT. Assuming that the miRNA profile reflects the cancer microenvironment, it can be postulated that up-/downregulated miRNAs in LM and BM are derived from a metastatic cancer cell’s response to the CNS micro-environment that differs from the primary site. The pathogenesis of LM remains largely unknown, but pre-existing parenchymal BM is a significant risk factor for developing LM based on the proportional incidence of LM in patients with BM and the development of LM after surgical spillage of BM [42]. In this study, most of (88%) the miRNAs that dysregulated in BM were also up-/downregulated in LM. This possibly means that these common miRNAs between LM and BM were metastatic cancer cells’ activity to adapt to the CNS microenvironment and other miRNAs, which discriminate LM from BM, and could explain the cancer cell adaptation to harsh aqueous CSF, which lacks nutrients and adhesion molecules and displays many biological characteristics of LM. Among these discriminative miRNAs of LM, miR-335-3p and miR-34b-3p were confirmed in their higher expression in LM compared to BM with the validation set. In addition, miR-335-3p had the largest number of target genes, which were stringed by up-/downregulated miRNAs in LM.

### 4.4. Limitations and Future Directions

Our LM and BM patients had heterogeneous primary cancers, and also BT patients had glioma and non-glial tumors with different cellular origins. Thus, our identified miRNA profiles may be affected by a different primary tumor histology. Although we verified that in a primary cancer of NSCLC, 92% of upregulated miRNAs in LM samples compared with BM samples were also found to be upregulated in comparison with all LM and BM samples, we need to limit the primary cancer type in future studies with a high enough number of samples to minimize miRNA profiles’ differences from various clinical factors. We are preparing a miRNA profiling analysis between the BM tissue and paired CSF to identify the source of discriminative extracellular miRNAs, but obtaining a comparable number of paired samples will take time.

In previous studies, CSF profiles differ according to the sampling site (ventricular vs. lumbar), especially in LM [24,43,44]. We could not control the sampling site (only tumor status), and we could not analyze paired samples (i.e., ventricular-lumbar) or subgroup differences according to the sampling site in this study due to a limited number of samples.

Another caveat of our study is lack of a functional study to verify miRNA target genes. Actually, we expect CSF proteomics to reveal the up-/downregulated expression of target proteins, but we could not find a definite accordance between CSF miRNA and their target proteins from mass-spectrometry-based proteomics with intermediate results from fewer than 10 samples (data not provided). We expect that more valid miRNA profiles from a larger number of samples and paired sample CSF proteomics will solve this problem.

## 5. Conclusions

We showed that miRNA profiling by miRNA microarray is a feasible method to identify CNS tumor status using a limited volume (2 mL) of CSF. We also determined the RNA yield with miRNA compositions of extracellular miRNAs in CSF according to patient groups. The miRNA profiles identified using comparisons between LM and BM samples suggest that these miRNAs are possibly derived from floating cancer cells and might be responsible for LM development, which in future studies could possibly be used as a biomarker for LM.

## Figures and Tables

**Table 1:** Clinical characteristics of cerebrospinal fluid sample analysis.

**Discovery Cohort (*n* = 65)**						
**Groups**	**Total**	**Cancer Control** **(*n* = 11)**	**Healthy Control** **(*n* = 12)**	**LM** **(*n* = 22)**	**Brain Metastasis** **(*n* = 11)**	**Brain Tumors** **(*n* = 9)**
Gender						
Male	29	5	4	13	3	4
Female	36	6	8	9	8	5
Median age (range)	56 (2.3–78)	9.0 (2.3–78)	61 (49–72)	57 (36–71)	47 (33–68)	30 (10–76)
Primary disease (*n*)		Leukemia (4) Lymphoma (2) Breast ca. (1) Cholangioca. (1) Chordoma (1) GCT (1) Melanoma (1)	Unruptured An. (10) Hydrocephalus (2)	NSCLC (16) Breast ca. (4) Ovarian ca (2)	NSCLC (5) Melanoma (3) Breast ca. (2) HCC (1)	Glioma (3) Schwannoma (2) HBL (1) Meningioma (1) Pitu. adenoma (1) Chondrosarc. (1)
Sampling site (*n*)						
Cisternal Lumbar Ventricular		0 11 0	10 2 0	0 12 10	2 9 0	1 6 2
**External Validation Cohort (*n* = 23)**						
**Group**	**Total**	**Healthy Control** **(*n* = 6)**	**LM** **(*n* = 11)**	**Brain Metastasis** **(*n* = 6)**		
Gender						
Male	10	2	7	1		
Female	13	4	4	5		
Median age (range)	65 (39–73)	59 (45–73)	69 (63–71)	64 (39–66)		
Primary disease (*n*)		Unruptured An. (4) HTN (2)	NSCLC (11)	NSCLC (1) Breast ca. (5)		
Sampling site (*n*)						
Cisternal Lumbar Ventricular		2 3 1	0 6 5	2 3 1		

Abbreviations: An, aneurysm; GCT, germ cell tumor; HBL, hemangioblastoma; HCC, hepatocellular carcinoma; HTN, hypertension; LM, Leptomeningeal metastasis; NSCLC, non-small cell lung cancer.

**Table 2:** Top 10 significantly upregulated and downregulated extracellular microRNA in CSF from patients with CNS tumors compared to control.

	Rank	Group 2, LM		Group 3, BM		Group 4, BT	
		microRNA	FC	microRNA	FC	microRNA	FC
Upregulated	1	miR-4275	15.4	miR-466	12.3	-	-
	2	miR-466	13.4	miR-4275	11.8	-	-
	3	miR-98-3p	11.2	miR-98-3p	9.50	-	-
	4	miR-34b-3p	10.7	miR-34b-3p	6.73	-	-
	5	miR-3128	9.97	miR-3591-3p	6.22	-	-
	6	miR-3119	9.38	miR-3145-5p	5.73	-	-
	7	miR-3591-3p	8.64	miR-7847-3p	5.06	-	-
	8	miR-4536-3p	7.42	miR-3119	4.83	-	-
	9	miR-3145-5p	7.31	miR-656-3p	4.56	-	-
	10	let-7a-3p	6.81	miR-885-5p	4.40	-	-
Downregulated	1	miR-455-3p	0.29	miR-1298-3p	0.18	miR-4708-3p	0.42
	2	miR-3921	0.34	miR-4708-3p	0.22	miR-6868-5p	0.47
	3	miR-1298-3p	0.35	miR-3619-5p	0.22	-	-
	4	miR-1263	0.40	miR-628-5p	0.27	-	-
	5	miR-4797-5p	0.45	miR-4797-5p	0.32	-	-
	6	miR-548a-3p	0.48	miR-877-5p	0.33	-	-
	7	miR-4708-3p	0.48	miR-6868-5p	0.33	-	-
	8	miR-548ac	0.49	miR-4742-5p	0.33	-	-
	9	-	-	miR-1263	0.38	-	-
	10	-	-	miR-602	0.38	-	-

The full list of differentially expressed miRNAs of each CNS tumor group is described in Supplementary Table S2. Abbreviations: BM, brain metastasis; BT, brain tumor; FC, fold change; LM, Leptomeningeal metastasis.

**Table 3:** Representative differentially expressed extracellular microRNAs in CSF of leptomeningeal metastases compared to brain metastases.

	Rank	All LM vs. BM			NSCLC	
	microRNA	FC		microRNA	FC
Upregulated	1	miR-3124-5p	4.74	hsa-miR-602	2.62
	2	miR-3619-5p	4.25	hsa-miR-335-5p	2.52
	3	miR-8084	4.17	hsa-miR-3128	2.47
	4	miR-3201	4.14	hsa-miR-4536-3p	2.31
	5	miR-3128	3.89	hsa-miR-4445-3p	2.26
	6	miR-335-5p	3.71	hsa-miR-3619-5p	2.25
	7	miR-602	3.42	hsa-miR-3124-5p	2.18
	8	miR-877-5p	3.36	hsa-miR-3910	2.11
	9	miR-4445-3p	3.15	hsa-miR-635	2.06
	10	miR-4423-3p	3.11	hsa-miR-4708-3p	2.05
	11	miR-668-5p	2.95	hsa-miR-4423-3p	2.04
	12	miR-371b-5p	2.65	hsa-miR-548ap-3p	2.03
	13	miR-3119	2.63	hsa-miR-877-5p	2.03
	14	miR-4536-3p	2.49		
	15	miR-2277-5p	2.48		
Downregulated	1	miR-7847-3p	0.18		
	2	miR-1281	0.23		
	3	miR-455-3p	0.24		
	4	miR-6776-5p	0.43		
	5	miR-3921	0.49		
	6	miR-1234-3p	0.50		

The miRNAs in shaded background in NSCLC column were also significantly upregulated in all LM comparison to BM. Abbreviations: BM, brain metastasis; FC, fold change; LM, Leptomeningeal metastasis; NSCLC, non-small cell lung cancer.

## Data Availability

The microarray data that support the findings of this study are available from NCBI Gene Expression Omnibus (Accession: GSE138092). The data that support the findings of this study are available from the corresponding authors upon reasonable request.

## Supplementary Materials

The following are available online at https://www.mdpi.com/article/10.3390/jcm10214860/s1. Figure S1: Overview of extracellular miRNA from cerebrospinal fluid (CSF). Figure S2: The validation of CSF extracellular miRNA microarray data using the digital droplet polymerase chain reaction (ddPCR) and measurement of classification scores. Table S1A: The purity and yield of extracellular RNA extracted from CSF according to patient groups. Table S1B. Sampling site of CSF samples. Table S2A: List of differentially expressed miRNAs between each patients groups by significance analysis of microarrays. CC vs. HC. Table S2B: List of differentially expressed miRNAs between each patients groups by significance analysis of microarrays. LM vs. Control. Table S2C: List of differentially expressed miRNAs between each patients groups by significance analysis of microarrays. BM vs. Control. Table S2D: List of differentially expressed miRNAs between each patients groups by significance analysis of microarrays. BT vs. Control. Table S2E: List of differentially expressed miRNAs between each patients groups by significance analysis of microarrays. LM vs. BM. Table S2F: List of differentially expressed miRNAs between each patients groups by significance analysis of microarrays. LM vs. BT. Table S2G. List of differentially expressed miRNAs between each patients groups by significance analysis of microarrays. BM vs. BT.
