# Supplementary material for: Exploratory Profiling of Extracellular MicroRNAs in Cerebrospinal Fluid Comparing Leptomeningeal Metastasis with Other Central Nervous System Tumor Statuses
Source: J Clin Med. 2021 Oct 22;10(21):4860. doi: 10.3390/jcm10214860 (PMC8584800; doi:10.3390/jcm10214860)
Supplement: Supplementary file 1 [file jcm-10-04860-s001.zip › Supplementary Information.pdf]

Supplementary Information

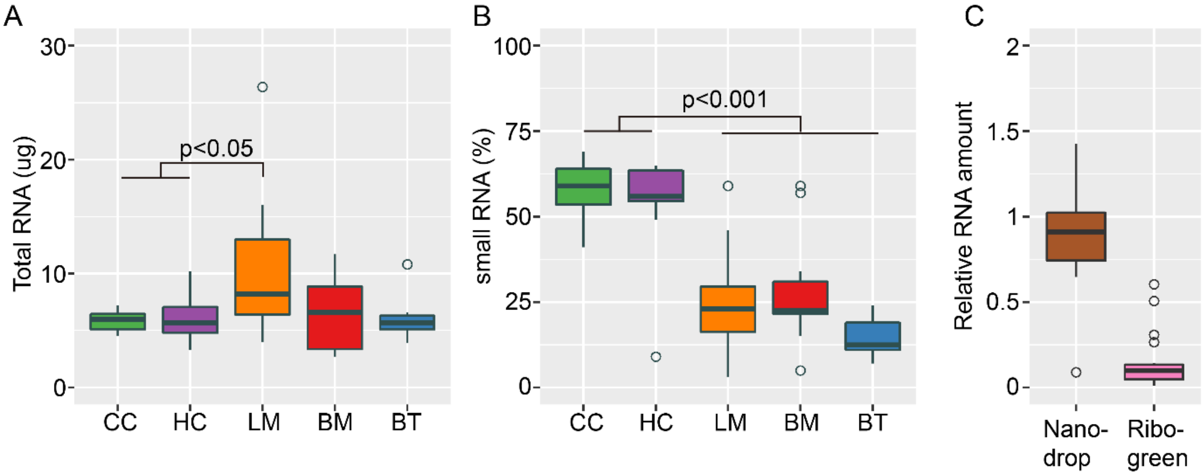

Supplementary Figure S1. Overview of extracellular miRNA from cerebrospinal fluid (CSF). (A) RNA yields isolated from 2 mL CSF in different patient groups were determined using Nanodrop^{TM} spectrometer. The mean RNA yield of patients with LM was significantly higher than that of other patient groups (ANOVA, Scheffe's test, $p<0.05$). (B) The miRNA percentage among small RNAs according to patient group measured by Agilent BioAnalyzer 2100TM. The control groups (CC and HC) showed significantly higher miRNA percentage than that of other groups (ANOVA, Scheffe's test, $p<0.001$). (C) The amounts of CSF extracellular RNAs were measured using Nanodrop and Ribogreen^{TM} assay. The amount of same sample differed according to measurement methods (n=8). Abbreviations: CC, cancer control; HC, healthy control; LM, leptomeningeal metastasis; BM, brain metastasis; BT, brain tumor. The box represents interquartile range and outliers were plotted in dots.

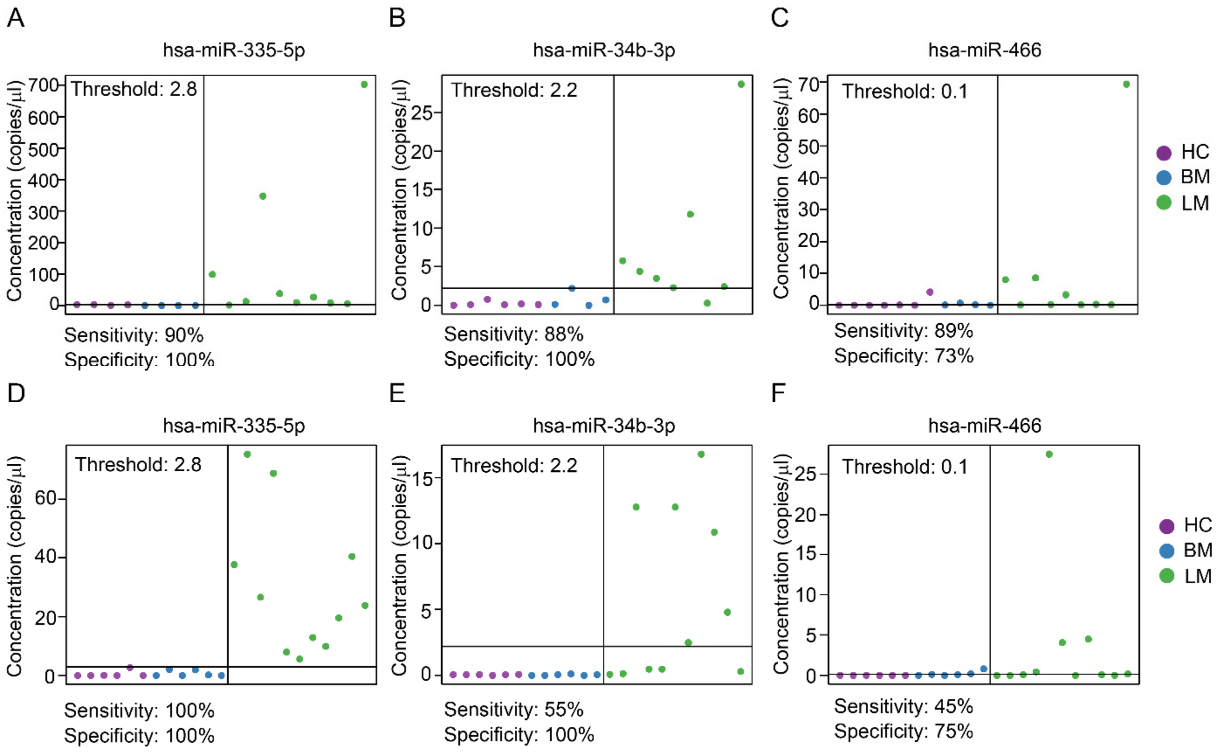

Supplementary Figure S2. The validation of CSF extracellular miRNA microarray data using the digital droplet polymerase chain reaction (ddPCR) and measurement of classification scores. (A-C) Expressions of indicated miRNAs were determined through ddPCR in the discovery sample set. Based on the concentration results from ddPCR, we measured sensitivity and specificity of the miRNA at the threshold, in which the sum of sensitivity and specificity was highest. (D-F) Expressions of indicated miRNAs were determined through ddPCR in the validation sample set. At the threshold from above, we measured sensitivity and specificity of the miRNA. The classification score was calculated using R software (version 3.6.0). Sensitivity=classified LM/ LM (%), Specificity=classified non LM/ non LM (%). Abbreviations: HC, healthy control; LM, leptomeningeal metastasis; BM, brain metastasis;
